# Population Structure, and Selection Signatures Underlying High-Altitude Adaptation Inferred From Genome-Wide Copy Number Variations in Chinese Indigenous Cattle

**DOI:** 10.3389/fgene.2019.01404

**Published:** 2020-02-14

**Authors:** Yaran Zhang, Yan Hu, Xiuge Wang, Qiang Jiang, Han Zhao, Jinpeng Wang, Zhihua Ju, Liguo Yang, Yaping Gao, Xiaochao Wei, Jiachen Bai, Yang Zhou, Jinming Huang

**Affiliations:** ^1^ Dairy Cattle Research Center, Shandong Academy of Agricultural Sciences, Jinan, China; ^2^ Key Laboratory of Agricultural Animal Genetics, Breeding and Reproduction of Ministry of Education & College of Animal Science and Technology, Huazhong Agricultural University, Wuhan, China; ^3^ Engineering Center of Animal Breeding and Reproduction, Jinan, China

**Keywords:** copy number variations, Chinese cattle, population structure, selection signatures, high-altitude adaptation

## Abstract

Copy number variations (CNVs) have been demonstrated as crucial substrates for evolution, adaptation and breed formation. Chinese indigenous cattle breeds exhibit a broad geographical distribution and diverse environmental adaptability. Here, we analyzed the population structure and adaptation to high altitude of Chinese indigenous cattle based on genome-wide CNVs derived from the high-density BovineHD SNP array. We successfully detected the genome-wide CNVs of 318 individuals from 24 Chinese indigenous cattle breeds and 37 yaks as outgroups. A total of 5,818 autosomal CNV regions (683 bp–4,477,860 bp in size), covering ~14.34% of the bovine genome (UMD3.1), were identified, showing abundant CNV resources. Neighbor-joining clustering, principal component analysis (PCA), and population admixture analysis based on these CNVs support that most Chinese cattle breeds are hybrids of *Bos taurus taurus* (hereinafter to be referred as *Bos taurus*) and *Bos taurus indicus* (*Bos indicus*). The distribution patterns of the CNVs could to some extent be related to the geographical backgrounds of the habitat of the breeds, and admixture among cattle breeds from different districts. We analyzed the selective signatures of CNVs positively involved in high-altitude adaptation using pairwise Fst analysis within breeds with a strong *Bos taurus* background (taurine-type breeds) and within *Bos taurus*×*Bos indicus* hybrids, respectively. CNV-overlapping genes with strong selection signatures (at top 0.5% of Fst value), including *LETM1* (Fst = 0.490), *TXNRD2* (Fst = 0.440), and *STUB1* (Fst = 0.420) within taurine-type breeds, and *NOXA1* (Fst = 0.233), *RUVBL1* (Fst = 0.222), and *SLC4A3* (Fst=0.154) within hybrids, were potentially involved in the adaptation to hypoxia. Thus, we provide a new profile of population structure from the CNV aspects of Chinese indigenous cattle and new insights into high-altitude adaptation in cattle.

## Introduction

Copy number variations (CNVs), which include insertions, duplications, and deletions of genomic segments among individuals within species, are currently known to be 50 bps to several Mbps in length (Mills et al., 2011; Iskow et al., 2012; Zarrei et al., 2015). CNVs encompass such more nucleotides than other types of variations (e.g., SNPs and InDels) that they can result in more remarkable effects on the functional gene through perturbing the long-range regulation of gene expression, altering gene dosage or coding sequences, and creating new genes, consequently contributing to phenotypic variations (Redon et al., 2006; Innan and Kondrashov, 2010; Bickhart and Liu, 2014; Shwan et al., 2017). A growing body of evidence shows that CNVs are crucial drivers of phenotypic diversity, evolution, and adaptation in humans and animals (Perry et al., 2008; Iskow et al., 2012; Sudmant et al., 2015; Romero et al., 2017; Rinker et al., 2019).

Cattle are economically and socio-culturally significant worldwide by supplying milk, meat, leather, and labor force (Taye et al., 2017). Since cattle have been domesticated, natural and artificial selection have been acting on the genome and changing the genome landscape of cattle breeds, leading to various environmental adaptation and agricultural traits (Taye et al., 2017). CNV, as an important source of genomic mutation, has been demonstrated to be widespread in cattle breeds worldwide (Liu et al., 2010; Hou et al., 2011; Choi et al., 2013; Sasaki et al., 2016; Upadhyay et al., 2017; Pierce et al., 2018; Rafter et al., 2018). Recently, CNVs have been increasingly shown to affect cattle traits of economic interest to humans, such as milk production (Xu et al., 2014), milk composition (Gao et al., 2017), residual feed intake (Zhou et al., 2018), body size (Zhou et al., 2016a), meat quality (Silva et al., 2016), and reproduction (Liu et al., 2019). Additionally, emerging evidence demonstrates that CNVs have been implicated in the adaptation of Nellore cattle to the tropical environment (Lemos et al., 2018). These findings indicate that artificial and natural selection may have shaped the landscape of CNVs in cattle genomes, thereby contributing to adaptive evolution, diversity, and breed differentiation.

In traditional Chinese culture, cattle symbolize diligence, honesty, responsibility, and selfless, with numerous ancient Chinese poetry and paintings in praise of cattle for their important contributions to agrarian society. China contains rich genetic resources of livestock, including yak (*Bos grunniens*), gayal (*Bos frontalis*), and indigenous cattle. A total of 53 indigenous cattle breeds have been recognized (National Bureau of Statistics, 2016), and analyses based on SNPs or microsatellites of the Y chromosome, mitochondrial DNA (mtDNA) sequences, and autosomal SNPs revealed the origins of indigenous cattle from *Bos taurus*, *Bos indicus*, and their hybrids (Lai et al., 2006; Li et al., 2013; Decker et al., 2014; Chen et al., 2018). Specifically, genome-wide SNPs reveal that there is a clear geographic clade regarding indicine admixture within Chinese cattle, greater in the southern and central regions but scarce in northwestern and northeastern China, as well as the Tibetan Plateau (Chen et al., 2018). Chinese indigenous cattle inhabit five different climatic zones, and exhibit phenotypic diversity and diverse environment adaptability. For example, Tibetan, Apeijiaza, and Shigatse Humped cattle inhabit the Tibetan Plateau, and these cattle show a superior tolerance to harsh climatic conditions, such as low pressure and low oxygen. Menggu, Fuzhou, and Hazake cattle reside in north, northeast, and northwest China, and they adapt well to cold winters. By contrast, some cattle breeds, such as Leiqiong, Ji’an, and Weizhou, tolerate the hot summers in south China. Accordingly, Chinese cattle breeds provide abundant genetic resources to understand the role of CNVs in the adaptation to a particular environment, especially to high altitude, to survive, maintain health, and reproduce.

Population relationships and high-altitude adaptation based on genome-wide CNVs have been analyzed in humans and yaks (Zhang et al., 2012; Sudmant et al., 2015; Wang et al., 2019). Moreover, a copy-number deletion associated with reduced blood concentrations of hemoglobin, a characteristic of high-altitude adaptation in Tibetans, has been identified (Lou et al., 2015). CNV data have been used to analyze population relationships in some Chinese indigenous cattle breeds (Zhang et al., 2015a; Mei et al., 2019). However, to date, high-altitude adaptation analyses of genomic CNVs in Chinese indigenous cattle are limited. Here, we selected 25 Chinese indigenous cattle breeds, as well as two yak populations as outgroups. We detected their genome-wide CNVs, and analyzed their population structure based on these CNVs. We used the pairwise Fst method to explore the selection signatures of CNVs positively involved in high-altitude adaptation, by comparing cattle breeds native to high-altitude plateau with those native to low-altitude land within taurine-type breeds and within *Bos taurus*×*Bos indicus* hybrids, respectively. The information gained in this study will be a valuable resource for understanding the role of CNVs in cattle evolution and breed formation, and will also provide new insights into the molecular mechanisms underlying high-altitude adaptation in cattle.

## Materials and Methods

### Ethics Statement

All experiments involving animals were conducted in accordance with the Regulations for the Administration of Affairs Concerning Experimental Animals published by the Ministry of Science and Technology of China in 2004 (http://www.most.gov.cn/fggw/zfwj/zfwj2006/200609/t20060930_54389.html). Our studies were approved by the Animal Care and Use Committee of the Dairy Cattle Research Center, Shandong Academy of Agricultural Sciences (Shandong, China).

### Blood Samples and Genotyping

**Figure 1:**
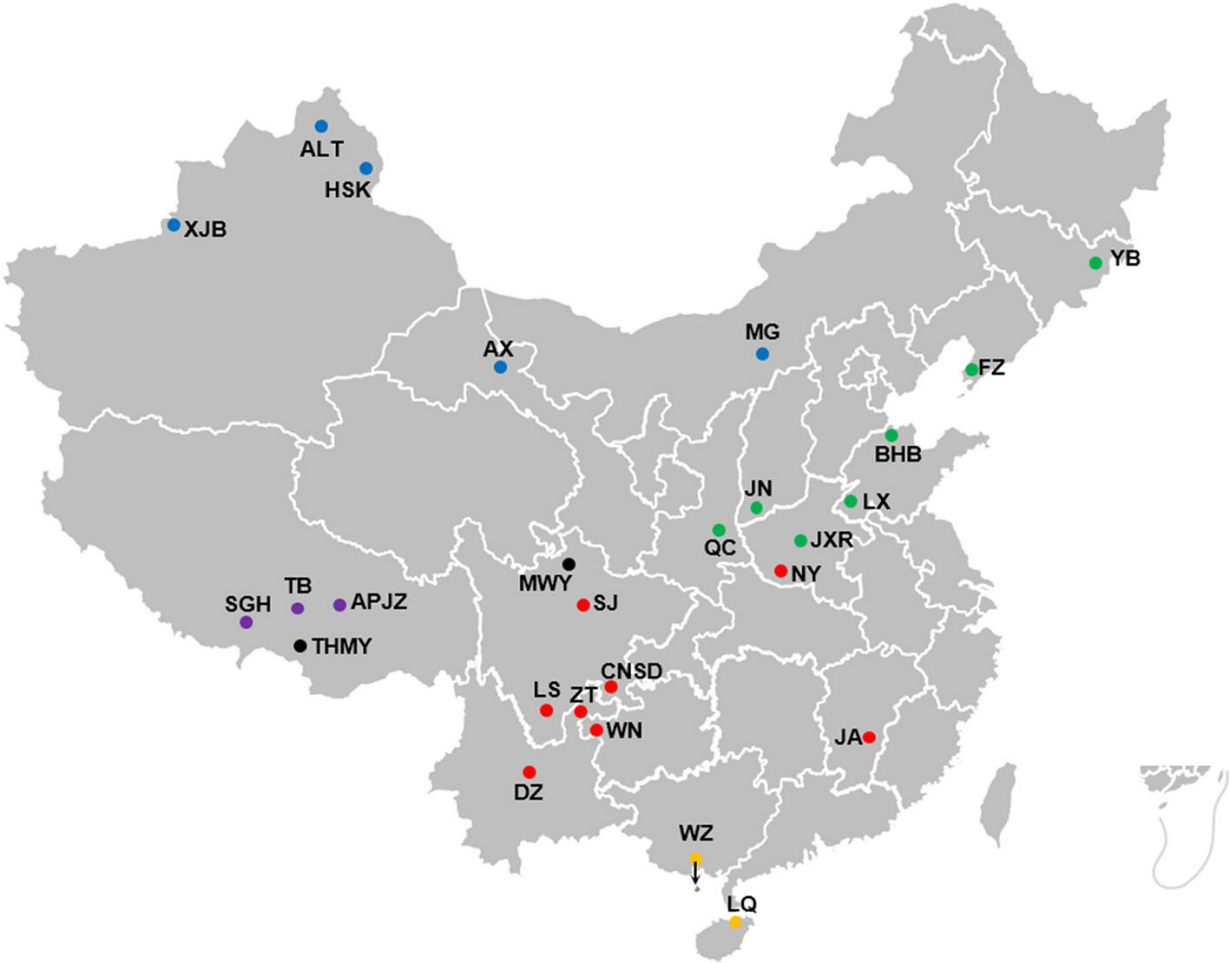
Geographic locations and climate of the habitats of Chinese cattle breeds. The solid dot represents each cattle breed, whereas the black solid dots represent outgroups, namely MWY and THMY. The solid dots in blue, green, purple, red, and orange represent temperate continental, temperate monsoon, plateau mountain, subtropical monsoon, and tropical monsoon climate, respectively.

Whole-blood samples were collected from 375 individuals representing 25 Chinese indigenous cattle breeds from five different climate zones across China (**Figure 1**): Aletai White cattle (ALT, n = 15), Anxi cattle (AX, n = 10), Apeijiaza cattle (APJZ, n = 12), Bohai Black cattle (BHB, n = 15), Chuannan Mountain cattle (CNSD, n = 13), Dianzhong cattle (DZ, n = 23), Fuzhou cattle (FZ, n = 11), Hazake cattle (HSK, n = 15), Ji’an cattle (JA, n = 20), Jiaxian Red cattle (JXR, n = 15), Jinnan cattle (JN, n = 15), Leiqiong cattle (LQ, n = 15), Liangshan cattle (LS, n = 15), Luxi cattle (LX, n = 15), Menggu cattle (MG, n = 11), Nanyang cattle (NY, n = 15), Qinchuan cattle (QC, n = 30), Shigatse Humped cattle (SGH, n = 13), Sanjiang cattle (SJ, n = 11), Tibetan cattle (TB, n = 15), Weining cattle (WN, n = 20), Weizhou cattle (WZ, n = 15), Xinjiang Brown cattle (XJB, n = 9), Yanbian cattle (YB, n = 11), and Zhaotong (ZT, n = 16). We also included 39 individuals representing two yak breeds (*Bos grunniens*) as outgroups, namely, Maiwa yak (MWY, n = 20) and Tibetan High Mountain yak (THMY, n = 19). Blood samples were immediately treated with an anti-coagulant. The detailed information about the habitats is supplied in **Supplementary Table S1**.

Genomic DNA was obtained using a standard phenol-chloroform protocol (Ausubel et al., 1993). Genotyping was performed using the Illumina BovineHD® Genotyping BeadChip (777,962 SNPs). Meanwhile, signal intensity data of each probe were also generated.

### Quality Filtering and CNV Calling

The samples with minor allele frequency of <0.02, call rate of <95%, and missing genotype frequency of more than 10% were excluded (Pierce et al., 2018). The PennCNV (Wang et al., 2007) was utilized to identify CNVs within the genome of cattle passing filtration. The PennCNV compile_pfb script was utilized to create a pfb (population frequency of B allele of SNPs) file from the data. The detect_cnv.pl was run to detect CNVs for 29 autosomes. The GC content within 1 Mb region (770 K per side) surrounding each marker was calculated and used to adjust the total signal intensity values of each SNP using the gcmodel option. We further filtered the low-quality samples with the following default thresholds: standard deviation (STD) of Log R Ratio (LRR) as 0.30, B Allele Frequency (BAF) drift as 0.008 and waviness factors as 0.08. The CNV regions (CNVRs) were determined by aggregating the overlapping CNVs identified across all samples and defined as three types (loss, gain, and both [loss and gain within the same region]) according to their composed CNV types (loss and gain). After filtering with the filter_cnv.pl script, a total of 355 individuals, including 318 indigenous cattle and 37 yaks, were used for further analyses (**Supplementary Table S1**).

### Gene Annotation and Ontology Analysis

We investigated the potential biological functions of the CNVRs detected in our study. The UCSC (https://genome.ucsc.edu/; Bos_taurus_UMD_3.1) database was used to annotate genes completely or partially overlapped by CNVRs. We performed gene ontology (GO) analysis using clusterProfiler package in R (3.5.3) (http://www.bioconductor.org/packages/release/bioc/html/clusterProfiler.html), with p-values adjusted by the Bonferroni method.

### Neighbor-Joining (N-J) Clustering Analysis and Principal Component Analysis (PCA)

The GenomicRanges Package (https://www.bioconductor.org/packages/release/bioc/html/GenomicRanges.html) in R (3.5.3) was used to portion the CNV to obtain the CNV segments according to the boundaries of the overlapped CNVs. We defined the genotype of CNV segments for each individual according to the type of unique CNV it belonged to as no overlap among unique CNVs in one sample. The python Sklearn module (https://scikit-learn.org/stable/) and the hclust function in R were used for PCA and N-J clustering analysis, respectively.

### Population Structure Analysis

STRUCTURE (v2.3.4) was used to conduct model-based clustering analyses to infer genome-wide ancestral admixture patterns of Chinese native cattle populations (Pritchard et al., 2000; Falush et al., 2003). Under admixture and allele frequencies correlated models, each analysis was run using 5,000 burn-in cycles and 50,000 MCMC (Markov Chain Monte Carlo) iterations. Inferred from the data at K = 2, multiple analyses with 10 independent runs for each K were performed with K increased from 2 to 12. The STRUCTURE output was submitted into STRUCTURE HARVESTER (http://taylor0.biology.ucla.edu/structureHarvester/) to infer the best K value (Earl and vonHoldt, 2012).

### High-Altitude Adaptation Analyses

To uncover the potential molecular mechanism for high-altitude adaptation of Chinese native cattle, we respectively performed selection signature analyses within taurine-type breeds (high-altitude TB versus low-altitude MG), and within *Bos taurus*×*Bos indicus* hybrids (high-altitude APJZ and SGH versus low-altitude BHB, LX, JXR and NY) using the pairwise Fst statistics. We calculated the Fst value for each CNV segment using the following equation as previously described (Poptsova et al., 2013):

$$Fst=\frac{Ht-Hs}{Ht};\;Ht\;=\;1-\sum \;ti^{2};\;ti\;=\;\frac{\left(xi\cdot Nx)+(yi\cdot Ny\right)}{\left(Nx+Ny\right)};\;Hs\;=\;\frac{(1-\sum xi^{2})\cdot Nx+(1-\sum yi^{2})\cdot Ny}{\left(Nx+Ny\right)};$$

where xi and yi are the population frequencies of allelic CNV segment number i (i = A0, A1, A2, A3, A4 or >A4) in population X and Y, respectively; Nx and Ny denote the number of individuals in population X and Y; and ti is the weighted average of xi and yi.

According to the Fst value, we chose the top 0.5% CNV segments to identify CNV-overlapping genes potentially related to high-altitude adaptation.

### Validation of CNVs by Quantitative PCR (qPCR)

Several CNVRs were randomly chosen for validation by qPCR. The bovine basic transcription factor 3 (*BTF3*) gene was used as the internal control. Primers (**Supplementary Table S2**) for qPCR were designed using Primer Premier 5.0 software (Premier, Canada). SYBR Premix Ex Taq II (TaKaRa, Dalian, China) was used for qPCR. Each sample (20 μL) was conducted in triplicate reactions on a LightCycler® 480 II (Roche) thermocycler following the instructions of SYBR Premix Ex Taq II (TaKaRa, Dalian, China). The results of relative expression were analyzed using the 2^-ΔΔCt^ method (Livak and Schmittgen, 2001). Given that cattle are diploid organisms, the copy number of 2 (cn = 2) detected by pennCNV was considered as the normal control, 0 (cn = 0) and 1(cn = 1) as losses, and 3 (cn = 3) to ≥4 (cn = 4) as gains.

## Results

### CNV and CNVR Discovery

A total of 375 Chinese indigenous cattle were selected for CNV detection. Quality control filtering resulted in the retention of 318 individuals (**Supplementary Table S1**). Fifty-seven individuals, including all of the sampled YB (n = 11), most of WZ (n = 10), several MG (n = 4), among others (**Supplementary Table S1**), were filtered out, which may be due to the low quality of DNA samples, or the low quality of chip data that may be related to ascertainment bias from the development of the commercial array. In total, 49,945 autosomal CNVs were detected, with an average of 157 CNVs found per individual (**Supplementary Table S3**). For each breed, the average number of CNVs ranged from 108 (CNSD) to 209 (LQ) (**Supplementary Table S3**).

A total of 5,818 CNVRs covering ~379.95 Mb sequences (**Supplementary Table S3**) were identified after the overlapping CNVs were merged into nonredundent CNVRs. These CNVRs corresponded to ~14.34% of bovine genome (UMD3.1 assembly), and included 2,872 deletions (losses), 742 insertions or duplications (gains) and 2,204 both events (**Supplementary Table S3**). For each breed, the CNVR coverage ranged from 1.70% (MG) to 3.81% (DZ) (**Supplementary Table S3**). The specific location of each CNVR on bovine chromosomes is presented in a vectorgraph (**Supplementary Figure S1**). Overall, the distribution of CNVRs throughout the bovine genome exhibited a non-random pattern. Chromosome 1 and 25 possessed the largest and the smallest number of CNVRs, respectively; the CNVR coverage ratio on chromosomes varied from 9.85% of chromosome 13 to 25.46% of chromosome 12 (**Supplementary Table S4**). The largest CNVR (4,477,860 bp in size, CNVR676) was found on chromosome 4, while the smallest (683 bp in size, CNVR4071) presented on chromosome 9 (**Supplementary Table S4** and **Table S5**). Moreover, the CNVR showed diversity among different breeds, and the chromosomal distribution of CNVRs (number, coverage ratio and size) across breeds varied greatly. For example, in ALT, the largest number of CNVRs was found on chromosome 6, and the coverage ratio ranged from 0.62% of chromosome 8 to 7.52% of chromosome 25, while in ZT, the largest number presented on chromosome 1 and the coverage ratio varied from 1.41% of chromosome 18 to 8.84% of chromosome 12 (**Supplementary Table S6**). For each breed, the statistical results of CNVR distribution on each chromosome are listed in **Supplementary Table S6**.

The accuracy of the CNVs was evaluated by qPCR with 70 samples of seven randomly selected CNVRs, and over 85% of the results were consistent with the detections by PennCNV (**Supplementary Table S7**).

### Gene Annotation and Ontology

A total of 2,189 of the 5,818 CNVRs overlapped 3,616 non-redundant genes (**Supplementary Table S5**). Among them, a total of 1345 genes have been previously reported as CNV-overlapping genes in Chinese indigenous cattle (Zhang et al., 2014; Zhang et al., 2015a; Zhang et al., 2015b; Xu et al., 2017; Yang et al., 2017; Mei et al., 2019) (**Supplementary Table S8**). As the Gene Ontology (GO) results were not significant after the P-values adjusted using the Bonfirroni method, which may be due to the genetic diversity of CNVRs among different breeds, we chose the top 100 GO terms for anaysis (**Supplementary Table S9**). GO analysis showed that these genes were implicated in many biological processes, and the most over-represented biological processes were responses to various stimuli or stresses (GO terms in bold-type letter in **Supplementary Table S9**) and regulations of different phases of the cell cycle (GO terms in italics in **Supplementary Table S9**).

### Population Relationships and Structure Based on Autosomal CNVs

#### N-J Clustering Analysis

Clustering analysis for individuals based on CNV segments was performed to obtain a global picture of group relationships. Results showed two main distinct branches, namely, branch one containing two yak groups (MWY and THMY) and branch two containing indigenous cattle breeds (**Supplementary Figure S2**). Among the indigenous cattle, LQ and partial JA (*Bos indicus*) could be separated, whereas the other indigenous cattle breeds exhibited highly intermixed clustering (**Supplementary Figure S2**).

#### PCA

**Figure 2:**
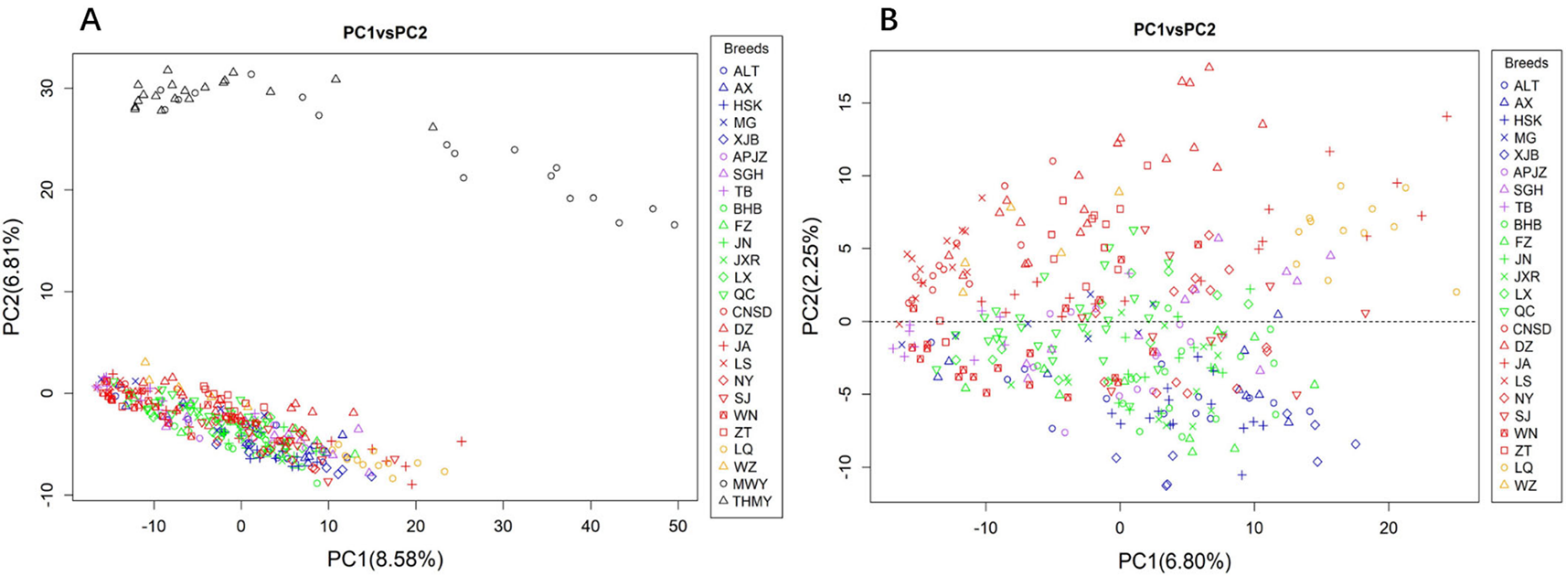
Principal component analysis (PCA) based on CNVs of animals assessed, with colors and shapes representing specific breeds. **(A)** PC1 and PC2 explain 8.58% and 6.81% of the total variations, respectively, separating the yak (*Bos grunniens*) from Chinese indigenous cattle breeds. **(B)** PC1 and PC2 capture 6.80% and 2.25% of the total variations of Chinese indigenous cattle, respectively, and distinguish the cattle breeds according to their geographic background.

We performed PCA of the autosomal CNVs genotype data. **Figure 2A** shows that the first two principal components (PCs), explaining 8.58% (PC1) and 6.81% (PC2) of the total variations, distinguished the yak populations (*Bos grunniens*), including MWY and THMY, from the Chinese indigenous cattle breeds, which was consistent with the result from N-J clustering analysis. We further performed PCA of indigenous breeds alone, because the great variation between the yak and the indigenous breeds could weaken the variation among indigenous breeds. **Figure 2B** illustrates that PC1 and PC2 captured 6.80% and 2.25% of the total variations of sampled Chinese indigenous cattle, respectively, and shows a separation of northwest-taurine type cattle from south-indicine and central-admixed type cattle, with most breeds not clearly differentiated. Specifically, cattle from northwestern China, including XJB, ALT, HSK, and AX, together with TB and MG, were mainly located below the dotted line (**Figure 2B**). Conversely, cattle from southern China, including LQ, JA, CNSD, DZ, ZT, and LS, were mainly located above the dotted line (**Figure 2B**). While the central-admixted type cattle, including NY, SJ, BHB, FZ, JN, JXR, LX, and QC, as well as APJZ and SGH from the Tibetan Plateau, showed intermixted location between cattle from northwestern and southern China. Most of the WN from southwestern China were closer to TB and cattle from northwestern China than those from southern China. **Supplementary Figure S3** displays PC3, which described 1.97% of the total variation, separated LQ and partial JA (*Bos indicus*) from other cattle breeds, in accordance with the N-J clustering results.

#### Admixture Analysis

**Figure 3:**
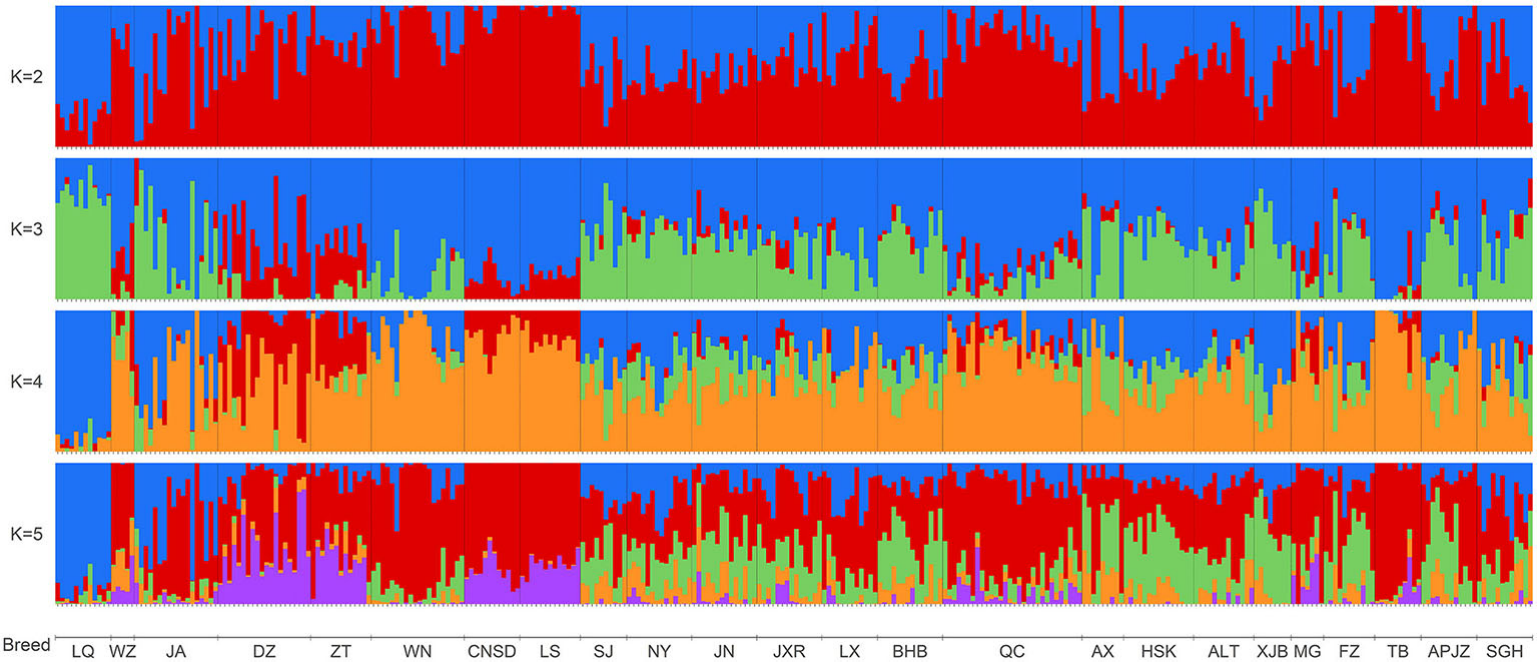
Bar plot presenting the population structure for 318 individuals from 24 Chinese indigenous breeds at assumed ancestry (K = 2 to K = 5). At K = 2, two ancestries, *Bos indicus* (blue) and *Bos taurus* (red), are distinguished.

We further elucidated the ancestral populations and the degree of admixture via structure analysis by increasing K from 2 to 12. Results from the STRUCTURE HARVESTER analysis suggested that K = 3 was the most likely number of genetically distinct groups within our samples (**Supplementary Figure S4**). Assuming K = 2, Chinese indigenous cattle breeds exhibited a mixture of *Bos taurus* (red color) and *Bos indicus* (blue color) with different degrees (**Figure 3**). When K was increased to the most likely number of three ancestral populations, genetic divergence within the zebu populations appeared, with LQ mainly harboring the so-called Chinese indicine type (green color), and some cattle breeds from southwestern China, including WZ, DZ, ZT, LS, and CNSD were influenced by another type of zebu, the Indian indicine type (red color) according to the results of Chen et al., 2018; TB clearly had taurine ancestry (blue color), and MG, QC, LS, CNSD, WN, and ZT were also mainly infuenced by the *Bos taurus* ancestry (**Figure 3**, **K = 3**). The cattle breeds from central China (NY, JN, JXR, LX, and BHB) together with APJZ and SGH from the Tibetan Plateau, and SJ were *Bos taurus*×*Bos indicus* hybrids (**Figure 3**, **K = 3**). Differences among individuals within the same population regarding these three ancestral groups were also observed.

### High-Altitude Adaptation Inferred From CNVs

We further identified the specific CNV or CNV-harbored genes with the potential to be positively involved in high-altitude adaptation, respectively within breeds with a strong *Bos taurus* background (high-altitude TB versus low-altitude MG), and within *Bos taurus*×*Bos indicus* hybrids (high-altitude APJZ and SGH versus low-altitude BHB, LX, JXR, and NY). We divided CNVs of individuals from both high and low altitudes into CNV segments according to the boundaries of individual CNV calls (Zhou et al., 2016b), and performed pairwise Fst analysis for each segment.

**Figure 4:**
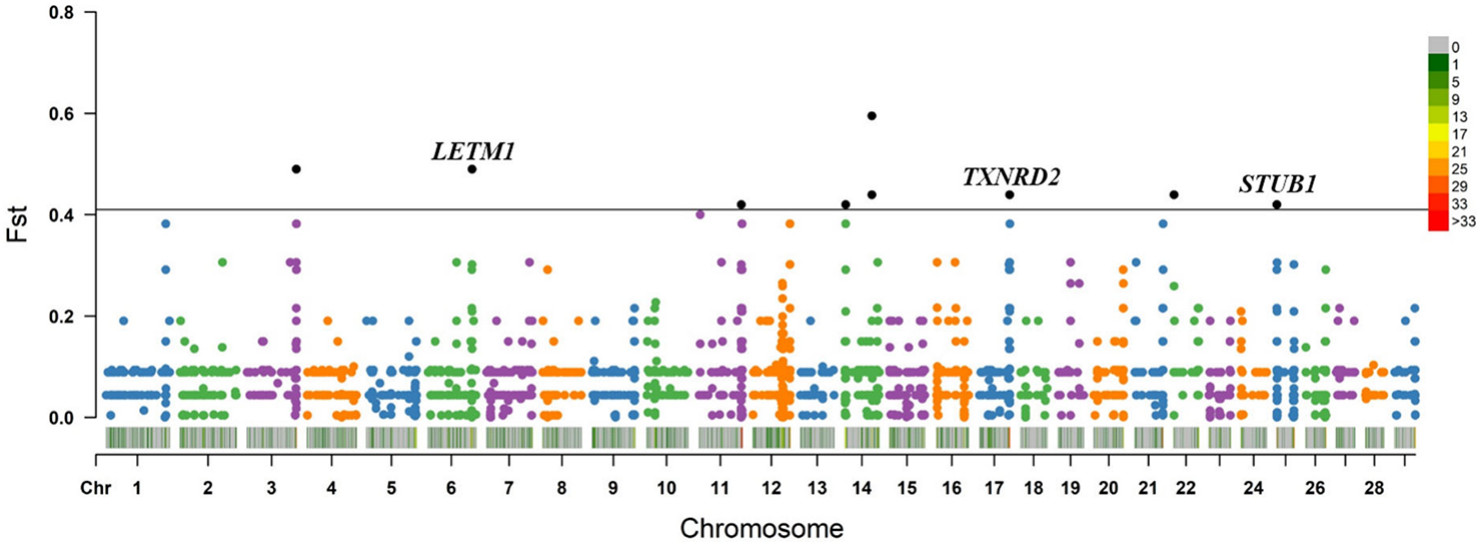
Fst value plots of genome-wide CNV segments within the taurine-type breeds (TB versus MG). The black line represents the top 0.5% threshold. The black solid dots represent the CNV segments at the top 0.5% of Fst value, with some dots overlapping. *LETM1, TXNRD2*, and *STUB1* have plausible biological functions related to hypoxic adaptation.

Within the taurine-type breeds, there were 11 CNV segments at the top 0.5% of Fst value (Fst ≥ 0.420, **Figure 4** and **Supplementary Table S10**), and we joined the connected segments into 9 CNV segments (**Table 1**). These CNV segments overlapped 15 functional genes, including *LETM1* (Fst = 0.490), *MGC148714* (Fst = 0.440), *TXNRD2* (Fst = 0.440), *SLC4A7* (Fst = 0.440), *SEPTIN5* (Fst = 0.440), *THEM6* (Fst = 0.420), and *STUB1* together with the other 8 genes within the same CNV segment (Fst = 0.420) (**Table 1** and **Supplementary Table S10**). The CNV segments overlapping *LETM1*, *SEPTIN5*, *TXNRD2*, *THEM6*, and *STUB1* exhibited higher frequency of loss of heterozygosity in high-altitude TB than in low-altitude MG (**Supplementary Table S10**). Conversely, *MGC148714* and *SLC4A7* displayed loss of heterozygosity only in MG in our study (**Supplementary Table S10**).

**Table 1:** List of top ranked CNV segments after joining connected ones and their overlapping genes within the taurine-type breeds (at top 0.5% level, UMD 3.1).

CNV segments	Overlapping genes
chr3:120552193-120556310	–
chr6:109709080-109714223	*LETM1*
chr11:103949579-104014308	–
chr14:2826632-2857000	*THEM6*
chr14:66636154-66666069	*MGC148714*
chr17:74805028-74836296	*SEPTIN5*
chr17:74895306-74905547	*TXNRD2*
chr22:1758241-1763355	*SLC4A7*
chr25:42521383-42561781	*RAB40C WFIKKN1 METTL26 RHOT2 LOC516108 STUB1 WDR24* *MCRIP2 FBXL16*

**Figure 5:**
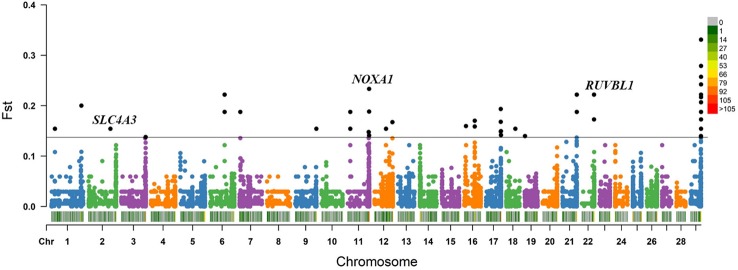
Fst value plots of genome-wide CNV segments within the *Bos taurus*×*Bos indicus* hybrid comparison (high-altitude APJZ and SGH versus low-altitude BHB, LX, JXR, and NY). The black line represents the top 0.5% threshold. The black solid dots represent the CNV segments at the top 0.5% of Fst value. *NOXA1*, *RUVBL1*, and *SLC4A3* have plausible biological functions implicated in hypoxic adaptation.

Within the *Bos taurus*×*Bos indicus* hybrid comparison, 43 CNV segments were identified at the top 0.5% of Fst value (Fst ≥ 0.138, **Figure 5** and **Supplementary Table S11**), and we joined the connected segments into 24 CNV segments (**Table 2**). These CNV segments also encompassed 15 functional genes, including *NOXA1* (Fst = 0.233), *RUVBL1* (Fst = 0.222), *SLC4A3* (Fst = 0.154), and so on (**Table 2** and **Supplementary Table S11**). The selected CNV segments overlapping *LOC524810* exhibited expanded copy numbers only in high-altitude cattle, whereas those overlapping the other 14 genes (*TOLLIP*, *IFITM1*, *IFITM3*, *NOXA1*, *SIGIRR*, *PKP3*, *RUVBL1*, *SSBP4*, *LRRC25*, *DYSF*, *COL4A1*, *SLC4A3*, *PRODH*, and *TALDO1*) showed higher frequency of reduced copy number in high-altitude cattle than in low-altitide ones (**Supplementary Table S11**). These findings suggest that the adaptation to high altitude may not only depend on a single CNV but a combination of various CNVs that were underwent strong positive selection in cattle.

**Table 2:** List of top ranked CNV segments after joining connected ones and their overlapping genes within the *Bos taurus*×*Bos indicus* hybrid comparison (at top 0.5% level, UMD 3.1).

CNV segments	Overlapping genes
chr1:16958282-16982945	–
chr1:147425142-147429091	–
chr2:108282665-108285133	*SLC4A3*
chr3:120415697-120425826	–
chr6:72002727-72106791	–
chr7:4676303-4705660	*SSBP4 LRRC25*
chr9:105024811-105035855	–
chr11:13001510-13026968	*DYSF*
chr11:103821456-103827010	–
chr11:105677940-105690535	–
chr11:105710194-105728308	*NOXA1*
chr12:57399771-57419504	–
chr12:88902827-88917105	*COL4A1*
chr16:8605747-8623933	–
chr16:51633917-51634954	–
chr17:73366927-73391908	–
chr17:74154357-74157145	*PRODH*
chr17:74292319-74305201	*PRODH*
chr19:1591038-1651670	–
chr21:71505398-71573501	*LOC524810*
chr22:60198815-60217277	*RUVBL1*
chr29:50824530-50830719	–
chr29:50854859-50864265	*TALDO1*
chr29:51174709-51371767	*SIGIRR PKP3 TOLLIP IFITM1 IFITM3*

## Discussion

### Abundant CNV Resources Were Detected in Chinese Native Cattle

CNVs, an important kind of genomic variations, are rife in cattle genome (Liu et al., 2010; Rafter et al., 2018). We successfully detected the genome-wide CNVs of 318 individuals from 24 Chinese indigenous cattle breeds. Despite previous studies have reported genome-wide CNVs in some Chinese cattle breeds (Zhang et al., 2014; Zhang et al., 2015a; Zhang et al., 2015b; Xu et al., 2017; Yang et al., 2017; Mei et al., 2019), our study is the first to detect the genome-wide CNVs of Chinese indigenous cattle breeds on a large scale, and the CNVs of 13 cattle breeds, including APJZ, ALT, CNSD, DZ, FZ, HSK, JA, SGH, SJ, TB, WN, WZ, and XJB, are first reported in current study. Although a low CNVR ratio was observed in each breed (from 1.70% to 3.81%), the total CNVR coverage reached ~14.34% of the reference (UMD3.1) in our study, more than the results reported previously in Chinese cattle (Zhang et al., 2014; Zhang et al., 2015a; Zhang et al., 2015b; Xu et al., 2017; Yang et al., 2017; Mei et al., 2019). Such abundant CNV resources might be attributed to multiple origins, diversity of geographical distribution and environments, and lack of systematic artificial selection of Chinese cattle. These CNV resources could facilitate the understanding of the evolution, phenotypic diversity, and evolutionary history of Chinese native cattle breeds.

### Genome-Wide CNVs Reflect the Population Relationships of Chinese Native Cattle

A growing body of evidences suggest that CNVs could provide important insights into the population structure and origins (Jakobsson et al., 2008; Berglund et al., 2012; Sudmant et al., 2015; Xu et al., 2016). The origin and genetic architecture of some Chinese cattle breeds have been previously investigated based on paternal (Y chromosome variations) (Cai et al., 2006; Li et al., 2013), maternal (mitochondrial DNA variations) lineage analyses (Lai et al., 2006; Lei et al., 2006; Jia et al., 2007), and genome-wide autosomal SNPs (Decker et al., 2014; Mei et al., 2017; Chen et al., 2018). Nevertheless, the present study is one of the first to attempt to explore the population relationships and genetic structure in a comprehensive analysis of Chinese cattle breeds on the basis of genome-wide CNVs derived from the high-density SNP array. Consistent with the mitochondrial DNA, Y chromosome and genome-wide SNPs analyses (Lei et al., 2006; Li et al., 2013; Xu et al., 2019), results of N-J clustering, PCA, and model-based structure analyses reflect the demographic events that shaped the genetic composition of Chinese cattle populations, and to some extent this includes some geographic structuring, but above all past admixtures between incidine and taurine lineages of various origins as described by Chen et al., 2018. These results based on the genome-wide CNVs support that Chinese cattle originate from *Bos taurus* and *Bos indicus*, as well as their hybrids, as previously reported (Decker et al., 2014; Chen et al.,2018). Moreover, our results are consistent with two kinds of zebu origins, as has been shown by the results based on SNPs (Chen et al., 2018). Unsurprisingly, ZT and DZ respectively from northeastern and middle Yunnan province influenced by *Bos taurus* and *Bos indicus* with ZT mainly influenced by the *Bos taurus*, in accordance with the results from mtDNA (Li et al., 2018). It should be pointed out that the ancestry admixtures of several breeds, such as LQ and JA, based on CNVs are not entirely consistent with the results based on SNPs, which may be due to that some portion of the total genetic vairation captured by CNVs is different from that by SNPs (Stranger et al., 2007), or due to the significant inter-individual and inter-breed differences of CNV (Mielczarek et al., 2018). Collectively, our results suggest that hybridization might have acted upon genomic CNVs and shaped the landscape of CNVs in Chinese cattle genomes, in consistence with the viewpoint proposed by Zhang et al 2015a.

### CNVs Revealed the Potential Molecular Basis to High-Altitude Adaptation

The Tibetan Plateau is known as the “roof of the world” with an average altitude above 4,000 m, where the climate is characterized by strong UV radiation, hypobaric hypoxia, and low temperatures. Oxygen (O_2_) plays essential role in animal’s metabolism, and it functions as the final electron acceptor during mitochondrial respiration which produces the vast majority of ATP at the last period of aerobic metabolism of glucose in cells (Taylor, 2008). Humans and animals native to high altitudes have to evolve certain adaptive mechanisms to offset the unavoidable environmental stress of hypoxia (Storz et al., 2010; Velotta et al., 2018). Studies have shown that maladaptive phenotypes about the circulatory systems that are related to the absorption and delivery of O_2_, such as increases in concentration of hemoglobin and erythrocytes, elevated pulmonary blood pressure (derived from vasoconstriction), and right-ventricle hypertrophy, are ubiquitous in low-altitude mammals when exposed to hypoxia (Tucker and Rhodes, 2001; Storz et al., 2010; Velotta et al., 2018), and low-altitude cattle are hyper-responders to high altitude (Tucker and Rhodes, 2001). Conversely, many high-altitude populations adapt well to hypoxia and exhibit a physiological reaction similar to low-altitude mammals with normal pulmonary blood pressure, right-ventricle size, and erythrocyte concentration, which can be referred to the effects of selection on genetically based trait variation that suppresses or eliminates these maladaptive phenotypes (Storz et al., 2010; Moore, 2017; Velotta et al., 2018). Hypoxia-inducible factors (HIFs) have been demonstrated to play a central role in the adaptive response to hypoxia by regulating expression of hypoxia-dependent genes that can increase systemic O_2_ delivery or improve cellular metabolic adaptation (Semenza, 2000; Covello and Simon, 2004; Taylor, 2008). Both in Tibetan and Andean highlanders, strong positive selections at genes in the HIF pathway have been identified as being involved in the hypoxic adaptation via genomic survey of SNPs (Bigham et al., 2009; Bigham et al., 2010; Xiang et al., 2013).

Previous results in other species showed that CNVs were major driving forces in adaptation evolution, especially during rapid evolution (Hastings et al., 2009; Iskow et al., 2012; Sudmant et al., 2015; Lauer et al., 2018). Environmental changes can drive accelerated adaptation through stimulated CNV (Hull et al., 2017). To date, in cattle, the understanding of the mechanism of high-altitude adaptation is mainly derived from studies of yak, a famous native breed in the Tibetan Plateau (Zhang et al., 2016; Wang et al., 2019). Chinese indigenous cattle inhabit more extensive altitudes than yak, from low-altitude coastal regions to the Tibetan Plateau, and may provide a better model to explore the genetic basis of high-altitude adaptation. The selective signatures of SNPs have been explored extensively in cattle (Pérez O’Brien et al., 2014; Xu et al., 2015; Zhao et al., 2015; Cheruiyot et al., 2018) and other livestock (Wilkinson et al., 2013; Onzima et al., 2018). However, similar analyses of CNVs are still limited. Positively selected CNVs or CNV-harbored genes in high-altitude cattle may play an important role in the adaptation to the harsh local environments. Thus, we first identified the selective signatures of CNVs positively involved in high-altitude adaptation using pairwise Fst analysis within taurine-type breeds (high-altitude TB versus low-altitude MG) and within *Bos taurus*×*Bos indicus* hybrids (high-altitude APJZ and SGH versus low-altitude BHB, LX, JXR, and NY), respcetively.

Within the taurine-type breeds, we identified 15 genes overlapped by the top 0.5% CNV segments. Among them, three genes (*LETM1*, *TXNRD2*, and *STUB1*) have plausible biological functions related to hypoxic adaptation (**Figure 4**). Over 90% of mammalian O_2_ is consumed through mitochondrial respiration (Rolfe and Brown, 1997). *LETM1* encodes the Leucine zipper/EF-hand-containing transmembrane protein 1(LETM1) embedded in the mitochondrial inner membrane, and it plays an essential role in maintaining normal mitochondrial morphology and cell viability (Schlickum et al., 2004; Li et al., 2019). Nevertheless, LETM1 overexpression can lead to decreases in mitochondrial ATP production and O_2_ consumption, mitochondrial dysmorphology, swollen mitochondria cristae, and an increase in fragmentation in Hela cells (Piao et al., 2009a; Piao et al., 2009b). In our study, a reduced copy number of the *LETM1* gene was discovered only in TB, implying that the reduced dosage of the *LETM1* gene may promote the adaptation to hypoxia by keeping normal mitochondrial morphology and respiration. *TXNRD2* encodes the mitochondrial thioredoxin reductase 2 (TXNRD2). Heart-specific TXNRD2-deficient mice showed reduced blood pressure, and stabilization of HIF-1α (Kiermayer et al., 2015). Additionally, overexpression of TXNRD2 attenuated NO-evoked accumulation and transactivation of HIF-1α in HEK293 cells (Zhou et al., 2008). STUB1, also known as CHIP, is an E3 ubiquitin ligase; it plays positive role in the ubiquitination and degradation of HIF-1α (Luo et al., 2010; Ferreira et al., 2013). Both *TXNRD2* and *STUB1* exhibited higher frequency of loss of heterozygosity in TB than in MG, implying that the reduced dosage of both *TXNRD2* and *STUB1* may benefit the adaptation to high altitude by mediating the stability and abundance of HIF-1α. Collectively, we found that CNVs possibly contributed to the hypoxia adaptation of TB through mediating mitochondrial morphology or function and stabilization of HIF-1α.

Within the *Bos taurus*×*Bos indicus* hybrid comparison, we also discovered 15 genes overlapped by the top 0.5% CNV segments. Among them, three genes (*NOXA1*, *RUVBL1*, and *SLC4A3*) have plausible biological functions implicated in hypoxic adaptation (**Figure 5**). *NOXA1* critically regulates NOX1-dependent superoxide production in atherosclerotic smooth muscle cells to drive plaque formation through smooth muscle proliferation, migration, and transition to a proinflamatory, macrophage-like phenotype (Orr and Woolard, 2019). Both global and smooth muscle-specific NOXA1 delection in ApoE knockout mice showed reduced plaque formation (Vendrov et al., 2019). *NOXA1* has been identified as a candidate gene for hypoxia adaptation in goat (Wang et al., 2016). In our study, the *NOXA1* gene showed higher frenquency of loss of heterozygosity in high-altitude cattle than in low-altitude cattle, which may benefit the health of cardiovascular system of high-altitude cattle. *RUVBL1* gene is implicated in hematopoietic stem cell survival (Bereshchenko et al., 2012), and heart growth and development (Rottbauer et al., 2002; Hartill et al., 2018). The expression of *RUVBL1* genes is significantly down-regulated in human mesenchymal stromal cells under short-term hypoxic stress (Udartseva et al., 2015). A reduced copy number of the *RUVBL1* gene showed a higher frequency in high-atitude cattle than in low-altitude cattle in our results, implying that the reduced dosage of the *RUVBL1* gene may benefit the adaptation to high altitude. *SLC4A3* encodes a plasma membrane Cl^-^/HCO_3_^-^ exchanger AE3, which belongs to the anion exchanger Solute Carrier Family 4 (SLC4) (Alper et al., 2002; Thorsen et al., 2017). The *SLC4A3* knockout mice exhibit normal respiratory response to changes in ambients O_2_ or CO_2_ but resistance to cardiomyocyte hypertrophy compared to wildtype littermates (Kampik et al., 2014; Sowah et al., 2014). The loss of heterozygosity at *SLC4A3* gene locus happened only in high-altitude cattle in current study, which may benefit in resisting the right-ventricle hypertrophy and maitaining healthy heart function under hypoxia. Taken together, we found that CNVs mainly promoted the hypoxia adaptation through influncing genes related to cardiovascular system in hybrid cattle.

## Conclusions

In this study, we determined the genome-wide CNVs for 318 animals from 24 Chinese native cattle breeds using the BovineHD Genotyping BeadChip array. A total of 5,818 nonredundant CNVRs covering ~ 379.95 Mbp (~14%) of the bovine genome were identified, providing abundant genomic variation resources of CNVs for Chinese cattle breeds. The distribution patterns of genome-wide CNVs could to some extent be related to the geographical backgrounds of the habitat, and admixture among cattle breeds from different districts. Furthermore, CNV-based ancestral admixture pattern analyses support that Chinese cattle originate from *Bos taurus* and *Bos indicus*, and their hybrids. CNV overlapping genes with high selective signals, such as *LETM1*, *TXNRD2*, and *STUB1* within taurine-type breeds, and *NOXA1*, *RUVBL1*, and *SLC4A3* within *Bos taurus*×*Bos indicus* hybrids, play potential important roles in the adaptation to high-altitude environments. Our results further enriched the role of CNVs in cattle adaptive evolution and breed formation. Thus, this study provides new insights into the population structure and the molecular basis underlying high-altitude adaptations. These results will constitute a valuable genetic resource for subsequent molecular breeding of Chinese native cattle. Future studies are required to explore the CNV structure using next-generation sequencing, and elucidate the functions and mechanisms of these CNVs in high-altitude adaptation. Future studies may obtain more insights on depicting the population structure of Chinese cattle through combining genome-wide SNPs and CNVs derived from the next-generation sequencing in larger populations.

## Data Availability Statement

The datasets generated for this study can be found in NCBI GEO GSE142218.

## Ethics Statement

The animal study was reviewed and approved by the Animal Care and Use Committee of the Dairy Cattle Research Center, Shandong Academy of Agricultural Sciences.

## Author Contributions

JH, XWa, and YZha conceived and designed the research. QJ, HZ, JW, ZJ, YG, XWe, and JB participated in the sample collection and DNA isolation. JH, YZ, YH, YZha, and XWa performed bioinformatics and statistical analyses. YZha drafted the manuscript, and YH participated in the methods writing. JH, YZ, and LY revised the manuscript. All authors read and approved the final manuscript.

## Funding

This research was supported by the Shandong Provincial Natural Science Foundation for Distinguished Young Scholars of China (JQ201709), the Natural Science Foundation of China (31771374), the Major Project of National Transgene in China (2018ZX08007001-002), the Agricultural Variety Improvement Project of Shandong Province (2019LZGC011), the Natural Science Foundation of Shandong Province of China (ZR2019BC051), the Fundamental Research Funds for the Central Universities (2662017QD016), and the Natural Science Foundation of Hubei Province of China (2018CFB363).

## Conflict of Interest

The authors declare that the research was conducted in the absence of any commercial or financial relationships that could be construed as a potential conflict of interest.
