# Supplementary material for: Population Structure, and Selection Signatures Underlying High-Altitude Adaptation Inferred From Genome-Wide Copy Number Variations in Chinese Indigenous Cattle
Source: Front Genet. 2020 Feb 14;10:1404. doi: 10.3389/fgene.2019.01404 (PMC7033542; doi:10.3389/fgene.2019.01404)
Supplement: Supplementary file 12 [file DataSheet_1.docx]

Supplementary Material

# Supplementary Figures

**
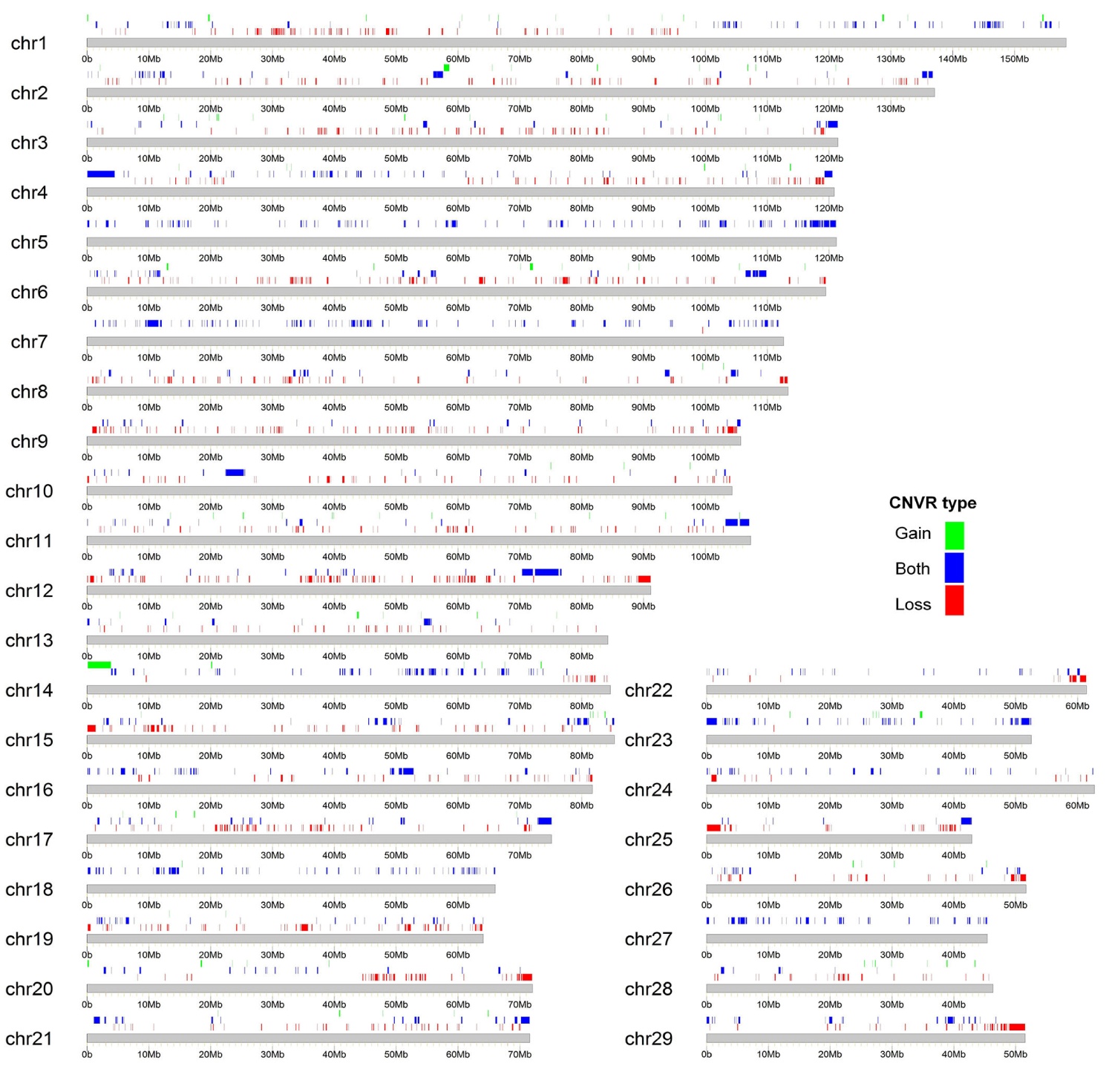
**

**Figure S1.** Genomic distribution of CNVRs in 318 Chinese indigenous cattle.


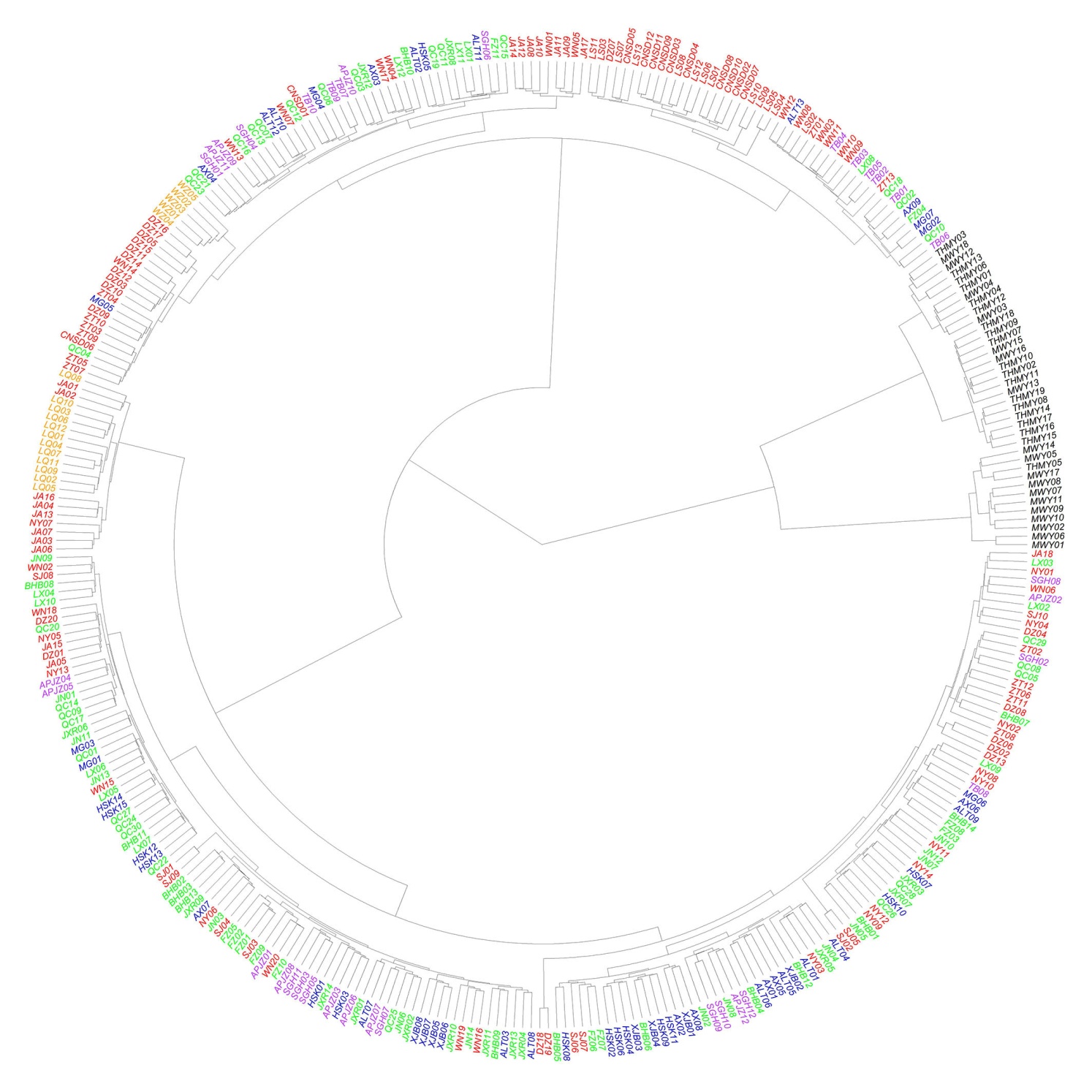


**Figure S2.** Neighbor-joining clustering map of 318 Chinese indigenous cattle and 37 yaks.

**
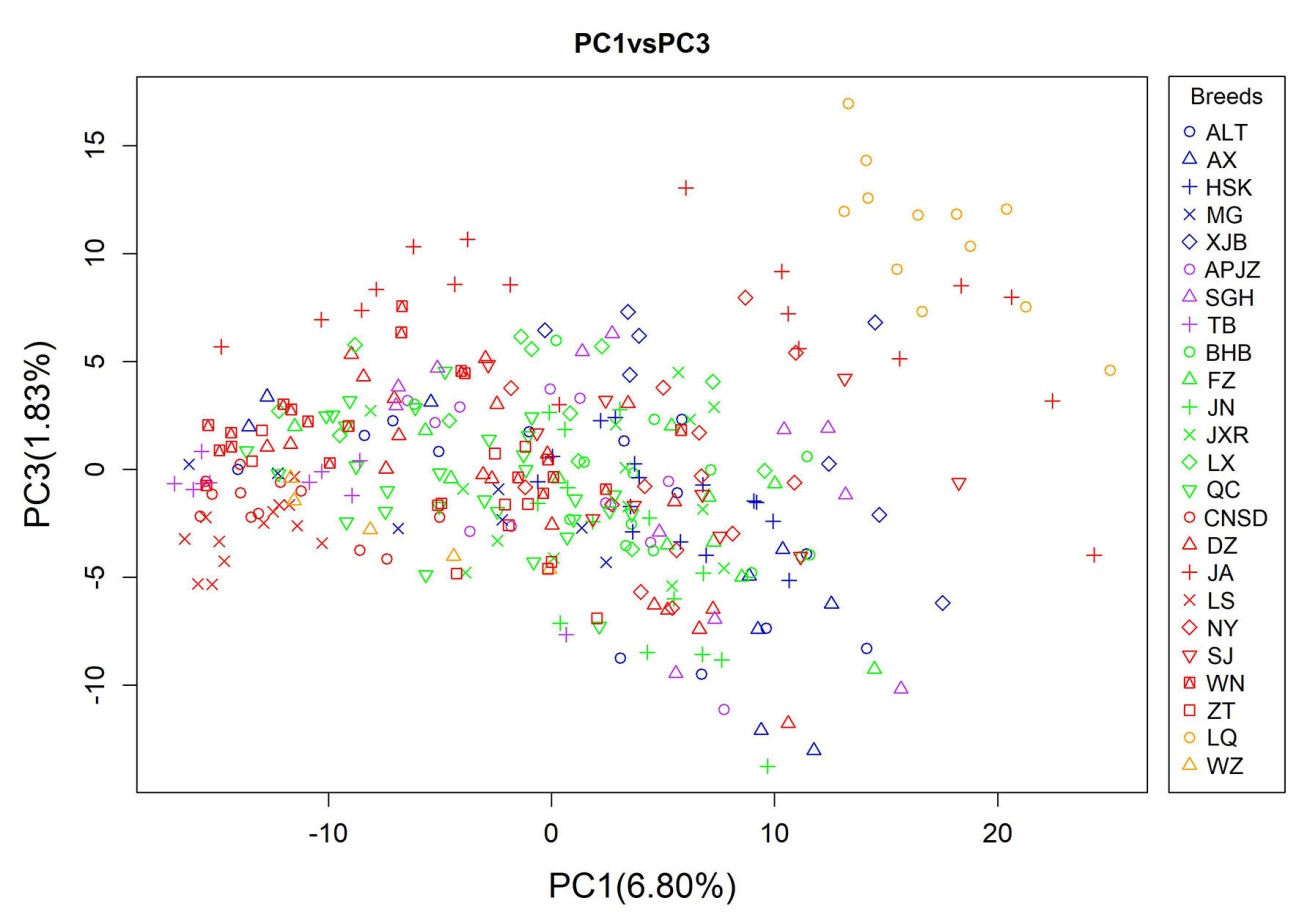
**

**Figure S3.** Principal component analysis (PCA) based on CNVs of animals assessed, with colors and shapes representing specific breeds. PC3 described 1.97% of the total variation, separated LQ and partial JA from other cattle breeds.


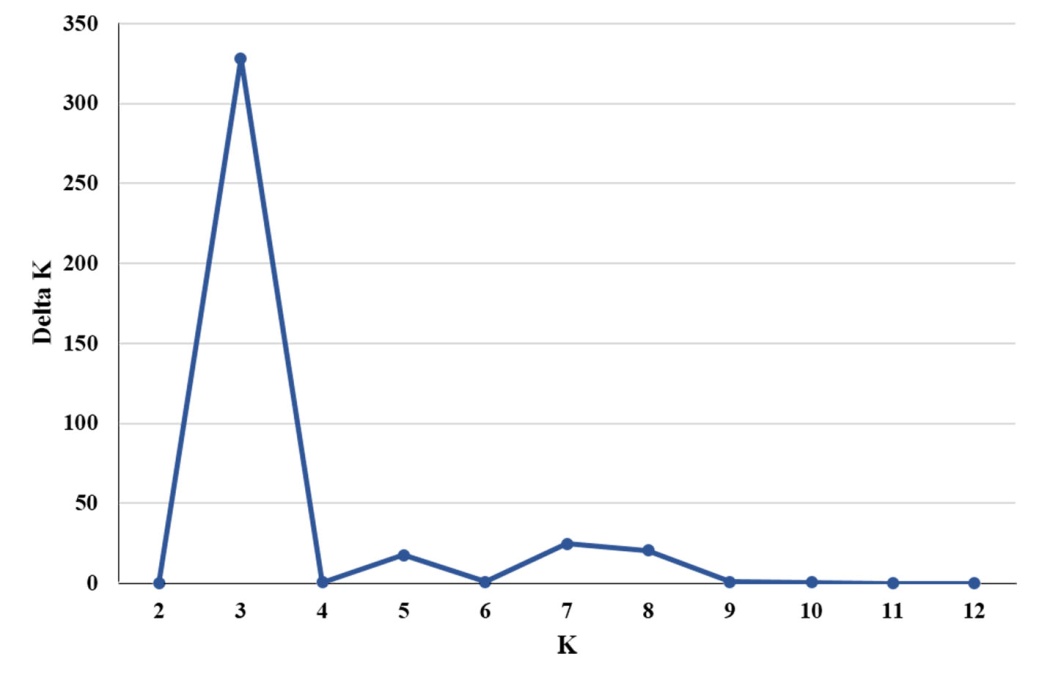


**Figure S4.** DeltaK values for K=2 to K=12. The DetaK value of K=3 is the largest. Accordingly, the best K value is the number of three.
